# Sequence information gain based motif analysis

**DOI:** 10.1186/s12859-015-0811-x

**Published:** 2015-11-09

**Authors:** Joan Maynou, Erola Pairó, Santiago Marco, Alexandre Perera

**Affiliations:** 1 Departament d’Enginyeria de Sistemes, Automàtica i Informàtica Industrial, Universitat Politècnica de Catalunya, Pau Gargallo, 5, Barcelona, 08028 Spain; 2 CIBER de Bioingeniería, Biomateriales y Biomedicina, Spain; 3 Institute for BioEngineering of Catalonia, balidiri Reixach 4-6, Barcelona, 08028 Spain; 4 Electronics Department in the University of Barcelona (UB), Martí i Franquès, 1, Barcelona, 08028 Spain

## Abstract

**Background:**

The detection of regulatory regions in candidate sequences is essential for the understanding of the regulation of a particular gene and the mechanisms involved. This paper proposes a novel methodology based on information theoretic metrics for finding regulatory sequences in promoter regions.

**Results:**

This methodology (SIGMA) has been tested on genomic sequence data for *Homo sapiens* and *Mus musculus*. SIGMA has been compared with different publicly available alternatives for motif detection, such as MEME/MAST, Biostrings (Bioconductor package), MotifRegressor, and previous work such Qresiduals projections or information theoretic based detectors. Comparative results, in the form of Receiver Operating Characteristic curves, show how, in 70 % of the studied Transcription Factor Binding Sites, the SIGMA detector has a better performance and behaves more robustly than the methods compared, while having a similar computational time. The performance of SIGMA can be explained by its parametric simplicity in the modelling of the non-linear co-variability in the binding motif positions.

**Conclusions:**

Sequence Information Gain based Motif Analysis is a generalisation of a non-linear model of the cis-regulatory sequences detection based on Information Theory. This generalisation allows us to detect transcription factor binding sites with maximum performance disregarding the covariability observed in the positions of the training set of sequences. SIGMA is freely available to the public at http://b2slab.upc.edu.

## Background

The information encoded in genetic sequences is expressed by means of a gene regulation process, which begins with a gene transcription step. The binding between specific proteins and their target sites in DNA is a key step in the control of the transcription process. These proteins – transcription factors (TF) – recognise specific motifs in DNA known as Transcription Factor Binding Sites (TFBS) or cis-regulatory sequences. The prediction, identification and detection of cis-regulatory sequences is a key factor in understanding gene regulation and in inferring regulatory networks [1, 2]. TFBS are usually very short (5 to 20 base pairs long) and highly degenerate, which gives rise to an extremely difficult identification problem due to low statistical power, as short sequences are expected to occur at random every few hundred base pairs. Due to their high variability, a consensus sequence approach for detection is insufficient. There is also evidence that this variability exhibits correlation between positions among the regulatory sequence [3, 4], and that this correlation could contain information which would help reduce the false positive rate and increase the sensitivity of a detector [5].

Due to the importance of identifying cis-regulatory sequences, much effort has been devoted to mapping the binding sites for a large set of transcription factors. An important recent project is the ENCODE (Encyclopedia of DNA Elements) project, which has been able to map 4 million regulatory regions in the human genome, opening new possibilities for computational methods [6]. Motif detection methods may be classified in different ways, depending on the approach adopted. Some reviews focus on the biology of motif discovery in regulatory regions [7, 8], whereas other publications focus more on the representation of the motifs: consensus-based methods and alignment-based methods [9]: consensus-based methods use word algorithms which consider binary hit/no-hit values [10, 11], and alignment-based methods use a set of alignment sequences with binding evidence to assign putative motifs to a candidate sequence. These latter methods could be classified as either numerical or stochastic models: numerical models are based on a mathematical representation of the nucleotides, whereas stochastic models, which are probably the most popular methods, are based on Position Weight Matrices (PWM) or Position Specific Weight Matrices (PSWM) [12]. A PWM is a matrix of scores corresponding to the frequency of the sequence symbols for each binding site position. The PWMs allow the capture of the variability over a sequence of nucleotides from a set of binding site positions [13], although there is the implicit assumption of independence between the residues of the aligned sequence matrix. PWM representations have been used in several algorithms to discover over-represented patterns from candidate sequences [14].

As noted above, statistical studies have shown the dependence among binding site positions variability. The common strategies for incorporating these dependencies within motif detectors include the extension of the PSSM approach to include pairs of correlated positions [15, 16], *m* ^*th*^ order Markov chains (HMM) [17, 18] and Bayesian Networks [19–22]. HMM can model the position interdependencies as long as high order HMMs, or a Bayesian approach are used but, in order to train any of both methods model sufficiently well, a huge training set of sequences would be required (± 1000 or more sequences per model).

A popular method, based on some of the previous work, is MEME/MAST, which provides an improved detection performance [23]. MAST is part of the MEME suite and uses a Q-FAST algorithm for finding motifs. Although these strategies may perform well in some datasets, they have shown certain limitations in the number of dependencies which may be considered between positions, in their ability to model dependencies between more distant positions, and in the large number of parameters which need to be adjusted in the models [3].

Previous work by our group proposed a parametric detector using the Rényi Entropy for binding site detection [24]. This measurement allowed us to build variable-sensitivity detectors modulated by the Rényi order – this assumed independence between binding site positions. A first approximation for modelling the correlation among binding site positions, known as Qresiduals, used a linear embedding to represent the set of binding site sequences [5] and employed a residuals-based approach as the detection statistic. Other non-related work modelled the pure correlation between binding site positions through non-linear correlations based on the variation of mutual information [25].

Statistical pattern recognition has also been applied to identification of sequence motif. Luo et al. [26] propose to use discriminant analysis for the prediction of Transcription Start Sites (TSS). From non-parametric measure, similar to Shannon information, Luo et al. [26] provide information about the variance observed in the dataset. This strategy has good performance for the binding motif detection when the motif positions are not correlated among them. But, this measurement does not allow modelling the dependencies among motif positions.

In this paper, we propose a generalisation of a non-linear model based on Information Theory, which allows modeling DNA contact by the protein and the biological interaction among binding sites using a small training set of sequences (5–50 sequences model). This new approach aims at a trade-off between the good generalisation properties of pure entropy methods and the ability of position-dependency metrics to improve detection power.

The performance of the proposed detector method, named SIGMA (Sequence Information Gain based Motif Analysis), is compared with different computational methods for binding site detection: MEME/MAST [23], Biostrings [27], MotifRegressor [28], Qresiduals [5] and a previously published set of algorithms based on information theory [24, 25].

## Methods

**Fig. 1:**
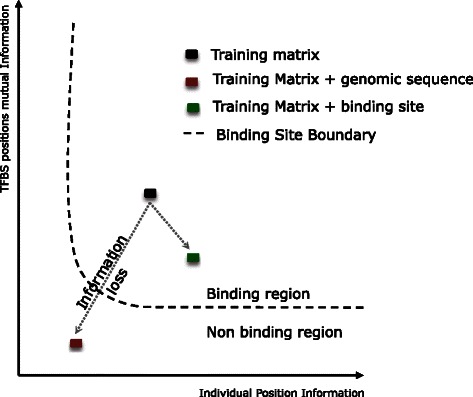
Information gain space defined by means of the variation on the information. X-axis on the graph shows the total amount of information change produced by assuming position independence. Y-axis shows the total amount of information change produced by assuming the correlation among positions. (*black square symbol*) Training matrix, (*red square symbol*) Training matrix with genomic sequence, (*green square symbol*) Training matrix with binding sites sequence. The broken line is the decision boundary

The information gain has been measured for each TFBS by means of two parametric uncertainty estimators. The rationale is based on the idea that the total information gain of a set of true TFBS aligned sequences will change according to the similitude of the new candidate sequence to that set (Fig. 1). The first estimator measures the total amount of information change produced by assuming position independence, whereas the second estimator measures the total amount of change of per-position mutual information (capturing pure correlation among binding site positions). Both estimators are computed by a parametric uncertainty measurement.

Let us consider a set of *I* aligned sequences (*s*_*i*_) with binding evidence *M*={*s*_*i*_,*i*=1,⋯,*I*}, and the same set including a candidate sequence $s_{c}, S = s_{c} \cup M$. Following Fig. 1, let *a* be the coordinate corresponding to the set *M*, with axes determined by the two measures previously mentioned. When a new candidate sequence is considered in *S*, both measures will vary to *b* or *g* depending on the nature of the candidate sequence. When the candidate sequence is a binding site sequence, (*b*,) the variation on the information will be not significant. However, when the candidate sequence is a genomic sequence, (*g*), the amount of information will vary significantly. With a sufficient training set, this information gain space can be split in two regions, genomic and binding, by means of a simple discriminant analysis which will define a decision boundary, as highlighted as a dashed line in Fig. 1. The decision boundary shape is the result of applying non-linear function.

### Information content measures

We have employed as parametric uncertainty measurements the Rényi entropy and Rényi Divergence (also called *α*-Divergence) [29], which are defined as:

(1) $$  H_{q}(X) =\frac{1}{1-q}{log_{2}{ \sum^{4}_{i=1} p(X_{i})^{q} }}  $$

(2) $$  D_{q}(X;Y) =\frac{1}{q-1}log_{2}{\sum^{4}_{i=1}\sum^{4}_{j=1}P\left(X_{i},Y_{j}\right)^{q}}Q\left(X_{i},Y_{j}\right)^{1-q}  $$

where *X*_*i*_ and *Y*_*j*_ are the nucleotides {*A*, *T*, *C* and *G*} at different DNA sequence positions, *P*(*X*,*Y*)=*p*(*X*,*Y*),*Q*(*X*,*Y*)=*p*(*X*)∗*p*(*Y*) and the *q* is the Rényi order which modulates the probability of occurrence of each symbol. *p*(*X*,*Y*) is the joint probability of *X* and *Y*, *p*(*X*) and *p*(*Y*) are the marginal probability. Both measurements (*H*_*q*_(*X*) and *D*_*q*_(*X*;*Y*)) depend on *q* which is a positive real number (*q*≠1)and both are non-negative for all *q*≥0. This parametrisation allows the building of a variable-sensitivity detector exploiting the statistical properties of the Redundancy, *R*, where *R* is defined as [24].

The measurement of the variation when the candidate sequence is added to the set has been computed using two heuristic functions, see (Eqs. 3 and 4). These functions depend on two parameters, *γ* and *ω*, which measure the difference between redundancies, Eq. 5, and divergence, Eq. 6, between the set of aligned sequences without the candidate sequences, *s*_*i*_, and with candidate sequence, *M*. These are estimated as described in Maynou et al. [24].

(3) $$  \rho (q,M) = \left|\sum_{i=1}^{L} R_{q}^{M_{i}} {\gamma}_{i} \right|^{-1}  $$

(4) $$  \eta(q,M)= \left|\sum_{i=1}^{L}|R_{q}^{M_{i}}| {\omega}_{i} \right|^{-1}  $$

where, *γ*_*i*_ and *ω*_*i*_ are

(5) $$  \gamma_{i}= \left|R_{q}^{M_{i}}-R_{q}^{S_{i}}\right|  $$

(6) $$  \omega_{i}= \left|D_{q}^{M_{i}}-D_{q}^{S_{i}}\right|  $$

where *L* is the number of nucleotides in the binding region, *M* is the aligned set of sequences with binding evidence and *i* is a specific column of *M*. ${R_{q}^{M}}$ is the redundancy, normalized depending on the maximum entropy on the set of aligned sequences, whereas ${R_{q}^{S}}$ contains the equivalent parametric entropy when the candidate sequence is assumed to belong to the set. The redundancy profile is a *L*-dimensional vector, where *L* is the total number of positions of the binding site. ${D_{q}^{M}}$ is the divergence matrix of the set of aligned sequences and ${D^{S}_{q}}$ is the divergence matrix considering the training matrix with the candidate sequence. The main diagonal is set to zero in each of these matrices, ${D_{q}^{M}}$ and ${D^{S}_{q}}$. The variation in the information is therefore calculated by means of *γ* and *ω* and *q*-values are optimised at the validation stage within the range (0,2]. As *q* increases, the noise included in the redundancy signal also increases [24]. From *q*-values higher than 2, signal-to-noise ratio is not optimal.

For a genomic sequence, the order of the system will decrease the values of *γ* and *ω*, whereas for a binding sequence the order of the system will not be altered substantially. Each candidate sequence will therefore be characterised by the pair (*x*=(*ρ*,*η*)) and classified as genomic or binding by means of a Quadratic Discriminant Analysis (QDA), as shown in Fig. 6. The decision boundary, *H*(*y*), is defined from the distribution of the variation on the information, *x*, for each class, genomic or binding, in the information gain space.

Binding site detection by means of the SIGMA algorithm can be summarized as follows, see Fig. 2:

1. Given a set of aligned sequences with binding evidence *M*, estimate the redundancy profile ${R^{M}_{q}}$ and the Rényi Divergence ${D_{q}^{M}}$ Eqs. (1) and (2).

**Fig. 2:**
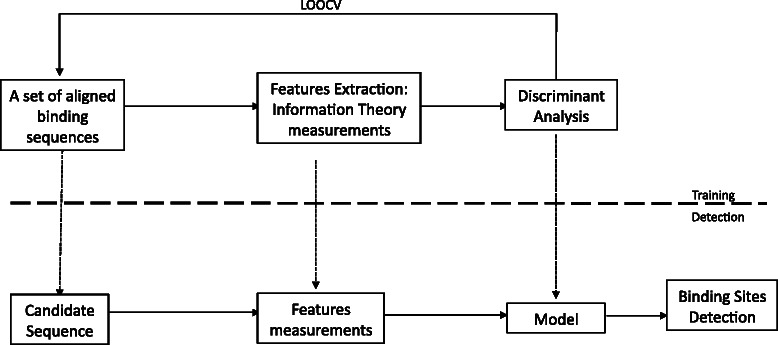
The essential steps in the training and detection process are shown for the SIGMA algorithm
2. Given a new candidate sequence, re-estimate both values assuming the candidate sequence belongs to *M*, ${R^{S}_{q}}$ and ${D^{S}_{q}}$,.
3. Compute the variation on the information *x*=(*ρ*,*η*) as defined in Eqs. (3) and (4).
4. Quadratic Discriminant Analysis is applied to the information gain space from the set of computed features.
5. Steps 3 and 4 are iterated over for each candidate sequence.

Additionally, for characterisation of the results we define a heuristic magnitude *C*, related to the *Complexity* of *M*, in order to characterise the degree of pure correlation between the variability of binding site positions in *M*, see (Eq. 7). *C* computes element by element the ratio between divergence value, where *D*_*q*_|_*i*,*j*_ is the element of *D*_*q*_ at row *i* and column *j*, and maximun entropy, *H*_*max*_ without to considerer the main diagonal. The average of the ratios define the complexity of *M*.

(7) $$  C= \frac{\sum_{i,j=1}^{N}D_{q}|_{i,j}}{N*(N-1)*H_{max}}\,;\quad i \neq j  $$

where *D* is the parametric uncertainty measurement considered, *N* is the size of the binding sites, *q* is the Rényi order and *H*_*max*_ is the maximum entropy for the set of probabilities *p*(*X*) and *p*(*y*), see section “Database description”. *C* is a value between 0 and 1. When *C* is close to 1, the degree of correlation among binding site positions is high.

### Database description

Data has been obtained from the Jaspar database [30], http://jaspar.genereg.net/ (see Tables 1 and 2).

**Table 1:** Summary of the transcription factors analysed for the *Homo sapiens* organism obtained from Jaspar database

TF	Family	Base	Sequences
*ELK4*	Ets	9	20
*ETS1*	Ets	6	40
*NFATC2*	REL	7	26
*MYCMAX*	bHLH	12	21
*E2F1*	E2F	8	10
*MAX*	bHLH	12	17
*NFIL3*	bZIP	11	23
*NFE2L2*	bZIP	11	20
*INSM1*	Zinc finger	12	24
*CREB1*	bZIP	12	16
*Irf2*	IRF	18	12
*FOXO3*	Forkhe	8	13
*HLF*	bZIP	12	18
*NFKappaB*	REL	10	38
*MZF114*	Zinc finger	6	20
*ESR1*	HNR	9	18
*FOXD1*	Forkhe	8	20
*MZF1513*	Zinc finger	10	16
*Ap1*	bZIP	7	18

**Table 2:** Summary of the transcription factors analysed for the *Mus musculus* organism from Jaspar database

TF	Family	Base	Sequences
*Pax2*	Homeo	8	31
*FOXO3*	Forkhe	8	13
*NFkappaB*	REL	10	38
*ARID3A*	ARID	6	27
*EBF1*	bHLH	25	10
*En1*	Homeo	11	10
*NR3C1*	HNR	18	9
*Egr1*	Zinc finger	11	15
*Ap1*	bZIP	7	18
*Runx1*	Runt	11	26
*CREB1*	bZIP	12	16
*AhrARNT*	bHLH	6	24
*Pdx1*	Homeo	6	31
*NFATC2*	REL	7	26
*Lhx3*	Homeo	13	20
*ARNT*	bHLH	6	20
*ELF5*	ETS	9	44

The *JASPAR Core* provides non-redundancy and high-quality alignment matrices for each transcription factor [30]. Results have been computed with background genomic sequences from the Eukaryotic Promoter Database (EPD) [31], using the EPD version based on the EMBL release 105 (sept 2010). The background loci chosen were *E* *P*74078(+)*H* *s* *R* *P* *S*9*P*2+ for *Homo sapiens* and *E* *P*07119(+)*M* *m* *I* *g* *k*0*M* *P* *C*11 for *Mus musculus*.

### Optimization

To apply SIGMA methodology to TFBS detection over genomic sequence, we should calculate the variation of the information, Eq. 4, as many times as the length of the sequence *I* (typically millions nucleotides). Given a sequence position, we must calculate the divergence between the binding site positions. This means that we must compute $\frac {L*(L-1)}{2}$ times the joint probability for each training matrix, where *L* is the total number of binding site positions in *M*. The running time of the algorithm depends on the length of the candidate sequence and on the number of binding site positions.The run time is therefore linear in the length of the input sequence and quadratic in the length of the binding site *L*.

(8) $$  T(L) \in O\left(L^{2}\right)  $$

The optimization algorithm is based on considering only the correlated binding site positions. The *η* function has been calculated considering only the Rényi-divergence of the correlated binding site positions (showing positive correlations) through a screening on the possible positive dependencies between these positions.

**Fig. 3:**
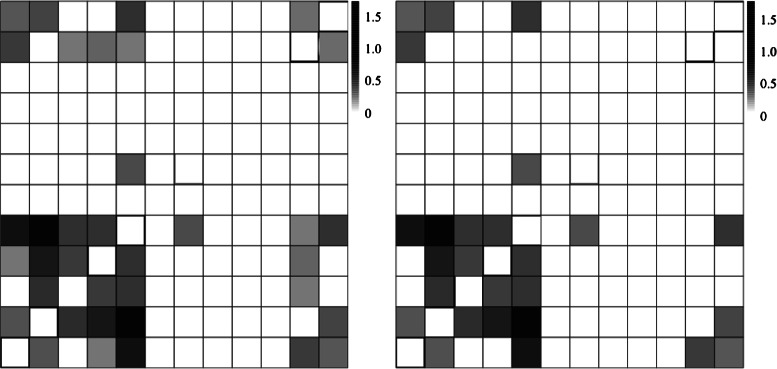
*Left*: Rényi Divergence, $D^{M}_{q=1}$, considering all possibles correlations between binding site positions. *Right*: $D^{M}_{q=1}$ considering only significant dependences between binding site positions after applying the error finite sample correction. Black boxes mean maximum correlation and white boxes mean zero correlation between binding site positions

Any two binding site positions are considered to be correlated if the Rényi divergence score is bigger than the error finite sample. This error yields to a bias on the uncertainty parametric measurement caused by estimating the probability using the nucleotide frequencies [24]. After the screening, we only compute based on the correlated positions of the training matrix as shown in Fig. 3.

**Fig. 4:**
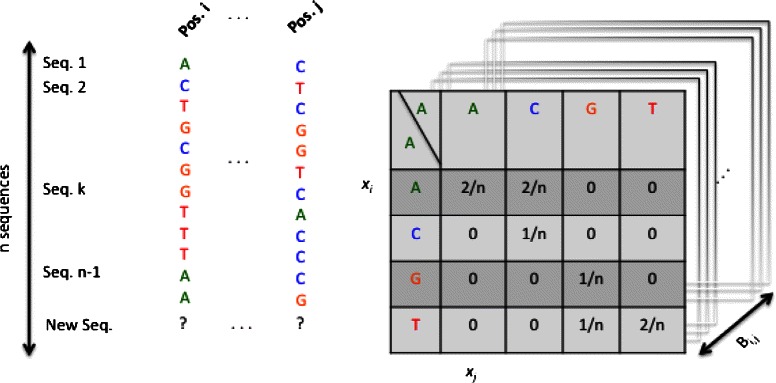
For each pair of positions *i* and *j* we calculate a joint probability matrix, $B_{i,j,x_{i},x_{j}}$, using all possible combinations of {*A*, *C*, *G* and *T*}

For each pair of positions (*i*,*j*) in *M* where *i*,*j*={1,…,*L*}, the joint probability for all the possible combinations of (*x*_*i*_,*x*_*j*_)= {*A*, *C*, *G* and *T*} are precomputed and stored in a 4×4 matrix. We construct a library $\left (B_{i,j, x_{i},x_{j}}\right)$ of sixteen 4×4 matrices containing all the possible joint probability values for each pair of positions *i* and *j* (as illustrated in Fig. 4).

For each new candidate sequence, we have to consider only the symbols matching correlated positions and read the joint probability value from the lookup table $B_{i,j,x_{i},x_{j}}$. The Rényi divergence and the discrimination function, *η* are then computed from these values. The estimated total number of significant transcription factor site dependencies in *Homo sapiens* and *Rattus novergicus* is approximately 50 *%* and 37 *%* [32]. In this way, the computing time can be reduced by approximately an order of magnitude.

### Validation

In order to build a model for each set of binding site sequences, the SIGMA detector has been characterized by means of leave-one-out cross validation (loo-cv). Each method has its own characteristic parameter. The range of the parameter used is different for each detector, see Table 3. The detector performance depends on the value of these parameters which have been selected employing loo-cv. Taking as a criteria a heuristic magnitude, *ν*_*auc*_. This parameter has been computed from the mean and variance of the area under the *N* ROC curve (*A* *U* *C*_*N*_) [5], which will be maximised for all methods.

(9) $$  \nu_{auc}= \mu_{auc}*(1-\sigma_{auc})  $$

**Table 3:** Summary of the characteristic parameters and the range considered for the validation of each computational method used

Method	Parameter	Range
*SIGMA*	Rényi Order	(0, 2]
*MEME/MAST*	Length Motif (L)	[1, L]
*Qresiduals*	Principal Components	[1, 10]
*Entropy*	Rényi Order	(0, 2]
*Divergence*	Rényi Order	(0, 2]
*Biostrings*	Not Applicable	Not Applicable
*MotifRegressor*	Length Motif (L)	[1, L]

where *μ*_*auc*_ and *σ*_*auc*_ are the mean and the variance of *A* *U* *C*_*N*_. *ν*_*auc*_ is a value between 0 and 1. When *ν*_*auc*_ is close to 1, the mean is close to 1 and the variance is close to 0.

From the performance data, we have calculated the mean and standard deviation of the AUC for each transcription factor and method by means of the outer loo-cv. This process has been repeated for all the TFs listed in Tables 1 and 2.

## Results and discussion

**Fig. 5:**
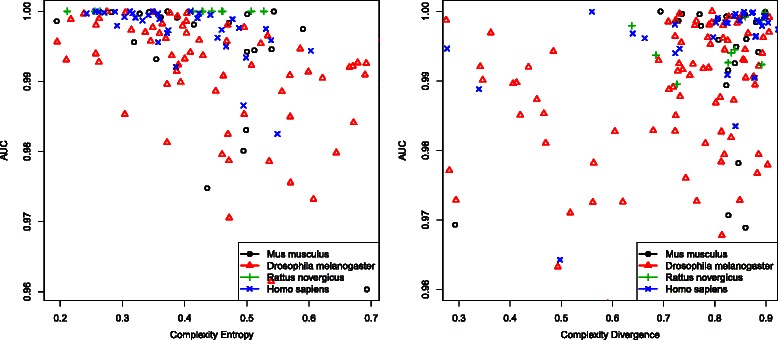
*Left* to *right*: Entropy and Divergence performances against *Complexity* (degree of correlation between binding site positions) for a set TF of different organisms ((*blue times symbol*) *Homo sapiens*, (*red triangle symbol*) *Drosophila melanogaster*, (*green cross symbol*) *Rattus norvegicus*, *(black circle symbol)* *Mus musculus*). Entropy performs better for low *Complexity*. On the contrary, Divergence performs better for large *Complexity*

We first show a characterisation of how the performance of the individual algorithms based on Entropy and Divergence depends on the complexity properties of the training matrix (*M*) *C*, (Eq. 7), see Fig. 5. The performance of these algorithms will vary on *C* depending on the design of each algorithm and the true correlation between positions found for each set of binding sequences. As one would expect, the total Entropy algorithm has a better behaviour with low values of *C*, whereas a Divergence based approach improves its performance when *C* is large. The SIGMA approach is partially based on both measurements and aims at finding a trade-off between both approximations in order to maximise the performance over the full dynamic range of *C*.

**Fig. 6:**
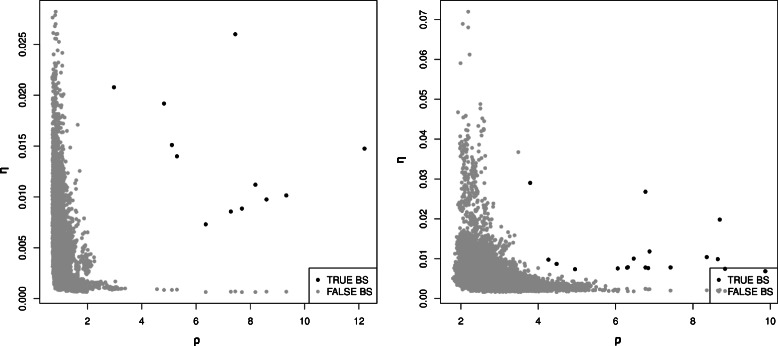
Empirical representation of the concept depicted in Fig. 1. *Left* to *right*: Information Gain when candidate sequences are inserted in the Transcription Factor Binding Sites *Irf2* and *HLF* for the *Homo sapiens* organism. Black points correspond to candidate sequences which are true binding site sequences. Grey points correspond to candidate sequences which are false binding site sequences

Figure 6 shows an example of real case where each input sequence is represented as a point in (*ρ*,*η*) coordinates. This set of samples includes genomic or binding sequences as shown in the figure. It is clear from the figure that both variables are contributing to the separation of the true binding site sequences.

**Fig. 7:**
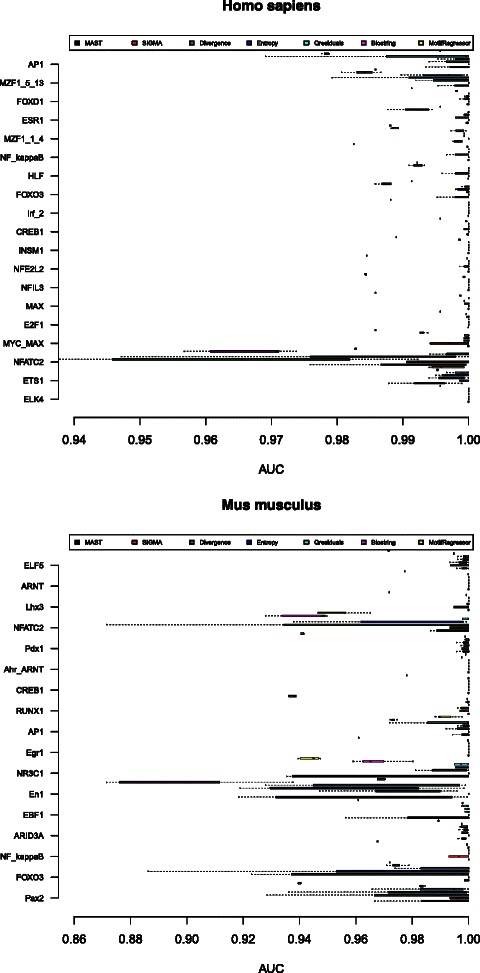
*Top* to *bottom*: Box plot of the AUC and its variation for the studied transcription factors for the *Homo sapiens* and *Mus musculus* organisms using different computational methods: (*black square symbol*) MAST, (*red square symbol*) SIGMA, (*green square symbol*) Divergence, (*blue square symbol*) Entropy, (*cyan square symbol*) Qresiduals, (*pink square symbol*) Biostring, (*yellow square symbol*) MotifRegressor. The background sequences used have been *E* *P*74078(+)*H* *s* *R* *P* *S*9*P*2+ for the *Homo sapiens* and *E* *P*07119(+)*M* *m* *I* *g* *k*0*M* *P* *C*11 for the *Mus musculus*

**Fig. 8:**
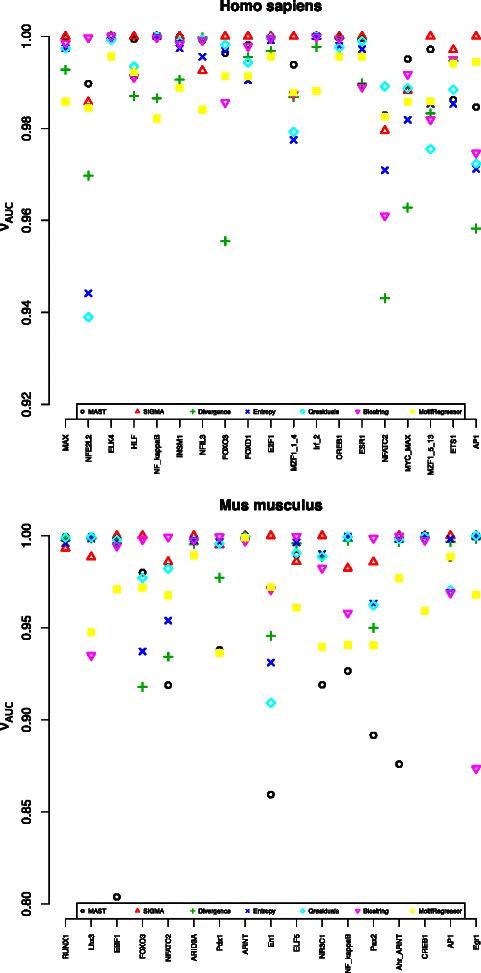
*Top* to *bottom*: Performance of each algorithm ((*black circle symbol*) MAST, (*red triangle symbol*) SIGMA, (*green cross symbol*) Divergence, (*blue times symbol*) Entropy, (*cyan diamond symbol*) Qresiduals, (*pink down-pointing triangle symbol*) Biostring, (*yellow diamond symbol*) MotifRegressor) is shown through *ν*_*auc*_, (Eq. 9), for a set of TFBS for the *Mus musculus* and *Homo sapiens* organisms. When *ν*_*auc*_ is close to 1, the mean is close to 1 and the variance is close to zero. For each TF, the best computational method will be that for which *ν*_*auc*_ is closest to 1

The performance of SIGMA, MEME/MAST, Qresiduals, Entropy, Divergence, Biostrings and MotifRegressor has been compared against the same set of TFs under the same validation conditions described in the previous section. In Fig. 7, it can be observed that the mean and standard deviation depend both on the Transcription Factor and on the method considered. The performance among all the methods has been compared by means of the *ν*_*auc*_ parameter described in Eq. (9). In Fig. 8, the *ν*_*auc*_ parameter is shown for each transcription factor and method. Based on the *ν*_*auc*_ values, in approximately 70 *%* of the TFBS under study, SIGMA shows better performance than the other methods. In 20 *%* of the TFs, the performance of the others methods is better than that of SIGMA. In the remaining cases, the SIGMA performance is similar to one or several of the computational methods considered. In most cases, the mean AUC is close to one and the variance is approximately zero, which suggests that SIGMA also behaves more robustly than other methods, as seen in Tables 4 and 5.

**Table 4:** Results for the set of computational methods considered for each TF of the *Homo sapiens* organism. The *ν*_*auc*_ is defined through the mean and variance of the *A* *U* *C*_*N*_ using a cross-validation method. Given a TF and method, *ν*_*auc*_ is chosen with maximum mean and lower variance in the *A* *U* *C*_*N*_

			*ν*_*AUC*_				
TFBS	MEME/MAST	Qresiduals	SIGMA	Entropy	Divergence	Biostrings	MotifRegressor
ELK4	0.99923	0.99993	1	1	0.99961	1	0.99566
ETS1	0.98621	0.98845	0.99707	0.98533	0.99473	0.99508	0.99415
NFATC2	0.98291	0.98915	0.97952	0.97091	0.94311	0.98284	0.98263
MYCMAX	0.9951	0.98872	0.98823	0.98187	0.96281	0.99178	0.98581
E2F1	0.99991	0.99963	1	0.99915	0.99685	0.99958	0.99566
MAX	0.99968	0.99743	1	0.99741	0.99275	0.99852	0.98583
NFIL3	0.9992	0.9994	0.99256	0.99558	0.999823	0.99917	0.98408
NFE2L2	0.98975	0.93901	0.98573	0.94418	0.96973	0.99974	0.9845
INSM1	0.99993	0.99891	1	0.99741	0.9906	0.99842	0.98885
CREB1	0.99965	0.99763	1	0.99793	0.99962	0.99953	0.99567
Irf2	0.99995	1	1	1	0.99773	0.99995	0.98817
FOXO3	0.99638	0.99817	1	0.99688	0.95549	0.98567	0.9915
HLF	0.99943	0.99343	1	0.99155	0.98706	0.99113	0.99216
NFkappaB	0.99987	1	1	1	0.98657	0.98256	0.98217
MZF114	0.99387	0.97925	1	0.97751	0.98682	0.98743	0.98775
ESR1	0.99962	0.99901	1	0.99725	0.98974	0.98903	0.9957
FOXD1	0.99814	0.99436	1	0.99043	0.99549	0.99787	0.99133
MZF1513	0.99719	0.97549	1	0.98534	0.9833	0.98193	0.98585
Ap1	0.98465	0.97231	1	0.97121	0.95825	0.97469	0.99445

**Table 5:** Results for the set of computational methods considered for each TF of the *Mus musculus* organism. The *ν*_*auc*_ is defined through the mean and variance of the *A* *U* *C*_*N*_ using a cross-validation method. Given a TF and method, the *ν*_*auc*_ is chosen with maximum mean and lower variance in the *A* *U* *C*_*N*_

			*ν*_*AUC*_				
TFBS	MEME/MAST	Qresiduals	SIGMA	Entropy	Divergence	Biostrings	MotifRegressor
Pax2	0.89161	0.96215	0.98572	0.96323	0.94998	0.98245	0.93971
FOXO3	0.98005	0.97719	1	0.93721	0.91796	0.97079	0.972
NFkappaB	0.92656	0.99944	0.982322	0.99949	0.99723	0.99939	0.96767
ARID3A	0.99757	0.99764	1	0.99771	0.99548	0.99753	0.98933
EBF1	0.80379	0.99787	1	0.9964	0.99593	0.99769	0.95929
En1	0.85943	0.90921	1	0.93119	0.94558	0.8736	0.96797
NR3C1	0.91904	0.98873	1	0.99017	0.98844	0.95811	0.94069
Egr1	0.99983	0.99996	1	0.99956	0.99826	0.99969	0.961
Ap1	0.98823	0.97044	1	0.99828	0.99672	0.96902	0.98861
Runx1	0.99937	0.99891	0.99323	0.99601	0.99743	0.99951	0.93645
CREB1	0.99997	0.99953	1	1	0.99958	0.99987	0.97698
AhrARNT	0.87593	0.99816	1	0.99828	0.99672	0.99721	0.99901
Pdx1	0.93796	0.99565	0.99499	0.99669	0.97722	0.99871	0.94051
NFATC2	0.91883	0.98219	0.98581	0.95394	0.934316	0.93503	0.9475
Lhx3	0.99961	0.99924	0.98846	0.99862	0.99852	0.9981	0.97183
ARNT	0.99998	0.99935	1	0.99945	0.99945	0.9999	0.9999
ELF5	0.98992	0.99045	0.98593	0.99641	0.99593	0.99453	0.97089

We computed a Wilcoxon rank-test [33] in order to estimate whether the improvement in performance is statistically significant. The null hypothesis was that the AUC distributions between SIGMA and other methods were the same and the alternative hypothesis was that the AUC distributions were different. The level of significance is represented by −*l* *o* *g*_10_(*p*_*value*_). Any *p*_*value*_>0.05 is shown in bold, see Tables 6 and 7). In most cases, it can be observed that the difference between the AUC distributions is significant.

**Table 6:** The level of significance corresponding to −*l* *o* *g*_10_ (*p*_*value*_) calculated using the Wilcoxon-rank test for the *Homo sapiens* organism. The null hypothesis is that the AUC distributions between SIGMA and the other computational methods are the same and the alternative hypothesis is that the AUC distributions are different. *p*_*value*_>0.05 is in shown in bold

			−*l* *o* *g*_10_(*p*_*value*_)			
TFBS	Qresiduals	MEME/MAST	Entropy	Divergence	Biostrings	MotifRegressor
ELK4	1.58	1.46	5.80	9.41	9.48	9.60
ETS1	3.48	7.55	7.96	7.52	7.51	7.85
NFATC2	**0.71**	7.61	2.81	5.21	9.48	9.59
MYCMAX	2.25	7.59	2.31	7.83	7.55	9.60
E2F1	1.58	7.12	2.33	3.12	7.56	9.6
MAX	3.73	4.16	2.66	5.13	5.10	6.46
NFIL3	**1.20**	6.10	**1.19**	6.05	6.21	7.82
NFE2L2	**1.20**	4.10	**0.80**	2.98	4.35	5.11
INSM1	2.33	8.63	**1.20**	2.08	8.95	10.11
CREB1	2.31	8.47	**1.20**	**1.20**	8.47	8.68
Irf2	**0.80**	6.79	3.37	6.14	6.78	6.89
FOXO3	2.31	6.11	5.63	5.20	6.48	8.26
HLF	3.38	4.45	**0.80**	**1.20**	2.08	6.02
NFkappaB	**1.20**	6.87	3.40	6.50	6.83	6.96
MZF114	7.52	13.95	10.99	3.90	14.11	9.65
ESR1	1.95	6.10	3.74	5.43	6.11	7.81
FOXD1	1.95	1.32	**1.20**	**1.09**	7.11	8.22
MZF1513	6.10	3.72	3.41	3.78	3.71	4.32
Ap1	4.75	13.51	2.67	3.03	13.5	17.14

**Table 7:** The level of significance corresponding to −*l* *o* *g*_10_(*p*_*value*_) calculated using the Wilcoxon-rank test for the *Mus musculus* organism. The null hypothesis is that the AUC distributions between SIGMA and the other computational methods are the same and the alternative hypothesis is that the AUC distributions are different. *p*_*value*_>0.05 is in shown in bold

			−*l* *o* *g*_10_(*p*_*value*_)			
TFBS	Qresiduals	MEME/MAST	Entropy	Divergence	Biostrings	MotifRegressor
Pax2	3.40	10.11	**0.81**	**1.20**	9.89	11.37
FOXO3	2.66	4.06	4.06	4.06	4.06	4.13
NFkappaB	7.14	8.80	5.65	4.88	9.13	11.08
ARID3A	10.05	2.68	**0.17**	**0.17**	2.68	9.5
EBF1	6.78	3.09	3.52	5.61	3.73	14.27
En1	4.06	4.82	2.66	5.10	5.10	6.47
NR3C1	3.37	5.79	**0.80**	**1.20**	4.53	7.14
Egr1	**1.20**	2.15	2.43	**2.14**	2.15	7.89
Ap1	4.75	4.76	2.66	4.76	4.76	4.89
Runx1	4.75	10.65	10.21	10.21	10.23	12.7
CREB1	1.57	3.71	3.01	2.66	3.71	3.72
AhrARNT	**1.19**	3.80	6.35	11.04	11.13	11.36
Pdx1	2.06	9.15	**0.80**	**0.80**	9.15	9.59
NFATC2	**0.21**	**0.66**	3.67	**0.05**	4.25	15.46
Lhx3	4.47	5.78	**0.80**	**0.80**	5.47	7.36
ARNT	**0.80**	**0.48**	**0.45**	1.78	**0.45**	11.28
ELF5	2.37	2.20	6.15	9.48	9.48	9.57

The computational time of SIGMA was compared with the set of computational methods considered. The C code for Qresiduals, Entropy and Divergence using the model obtained in validation and MEME/MAST (Version 4.4.0) was used and has been made publicly available. The run time was obtained in comparison with randomly generated candidate sequences of 1500 nucleotides. The total time has been calculated from 100 iterations of each algorithm. The averages of the computational times in detection for the set of TF considered of *Homo sapiens* (Tables 4 and 5) are shown in Table 8.

**Table 8:** Per CPU, the total run time was calculated on a 2.3 GHz Intel Core 2 Duo *P*8600 computer with 4 GB RAM

Method	Run time (s)	sd (s)
SIGMA	0.132	0.007
Qresiduals	0.119	0.006
Entropy	0.051	0.003
Divergence	0.081	0.004
MEME/MAST	0.019	0.001
Biostrings	0.004	0.0001
MotifRegressor	0.144	0.02

## Conclusions

A new methodology based on a discriminant analysis of two information theoretic measures has been proposed for binding site detection. The variation on the information has been measured through two parametric uncertainty measurements (the Rényi entropy and Rényi divergence). The method focusses on the variation in these information measures when a new sequence is assumed to belong to a training set of sequences with known binding properties.

This methodology allows us to detect cis-regulatory sequences with maximum performance disregarding the co-variability observed in the positions of the training set of sequences. SIGMA has been characterised on the detection problem for a large set of transcription factors and compared with different motif detection algorithms. AUC distributions have been calculated which show that there is a statistically significant difference between SIGMA performance and the performance of the other methods. In approximately 70 *%* of the cases considered, SIGMA has exhibited better performance properties, at comparable levels of computational resources, than the methods with which it was compared.

As you can see through the heuristic parameter, SIGMA method is more robust than the other methods. A model based on both parametric uncertainty measurements can be useful to detect cis-regulatory sequences. But when the number of the positions involved in the binding sites process is small, the SIGMA performance is comparable with the rest of the computational methods.

## Abbreviations

AUC: Area Under ROC Curve
ENCODE: Encyclopedia of DNA Elements
LOO-CV: Leave-one-out cross-validation
PWM: Position Weight Matrices
PSWM: Position Specific Weight Matrices
QDA: Quadratic Discriminant Analysis
ROC: Receiver Operating Characteristic
SIGMA: Sequence Information Gain Motif Analysis
TF: Transcription Factor
TFBS: Transcription Factor Binding Sites
TSS: Transcription Start Sites.
